# ISHAPE: new rapid and accurate software for haplotyping

**DOI:** 10.1186/1471-2105-8-205

**Published:** 2007-06-15

**Authors:** Olivier Delaneau, Cédric Coulonges, Pierre-Yves Boelle, George Nelson, Jean-Louis Spadoni, Jean-François Zagury

**Affiliations:** 1 Chaire de Bioinformatique, Conservatoire National des Arts et Métiers, 292 rue Saint-Martin, 75003 Paris, France; 2 Unité INSERM U736, Centre de Recherche des Cordeliers, 15 rue de l'École de Médecine, 75006 Paris, France; 3 Unité INSERM U707, 27 rue de Chaligny, 75012 Paris, France; 4 Laboratory of Genomic Diversity, SAIC-Frederick, MD, USA; 5 Unité INSERM U841, 8 rue du général Sarrail, 94000 Créteil, France

## Abstract

**Background:**

We have developed a new haplotyping program based on the combination of an iterative multiallelic EM algorithm (IEM), bootstrap resampling and a pseudo Gibbs sampler. The use of the IEM-bootstrap procedure considerably reduces the space of possible haplotype configurations to be explored, greatly reducing computation time, while the adaptation of the Gibbs sampler with a recombination model on this restricted space maintains high accuracy. On large SNP datasets (>30 SNPs), we used a segmented approach based on a specific partition-ligation strategy. We compared this software, Ishape (Iterative Segmented HAPlotyping by Em), with reference programs such as Phase, Fastphase, and PL-EM. Analogously with Phase, there are 2 versions of Ishape: Ishape1 which uses a simple coalescence model for the pseudo Gibbs sampler step, and Ishape2 which uses a recombination model instead.

**Results:**

We tested the program on 2 types of real SNP datasets derived from Hapmap: adjacent SNPs (high LD) and SNPs spaced by 5 Kb (lower level of LD). In both cases, we tested 100 replicates for each size: 10, 20, 30, 40, 50, 60, and 80 SNPs. For adjacent SNPs Ishape2 is superior to the other software both in terms of speed and accuracy. For SNPs spaced by 5 Kb, Ishape2 yields similar results to Phase2.1 in terms of accuracy, and both outperform the other software.

In terms of speed, Ishape2 runs about 4 times faster than Phase2.1 with 10 SNPs, and about 10 times faster with 80 SNPs. For the case of 5kb-spaced SNPs, Fastphase may run faster with more than 100 SNPs.

**Conclusion:**

These results show that the Ishape heuristic approach for haplotyping is very competitive in terms of accuracy and speed and deserves to be evaluated extensively for possible future widespread use.

## Background

Studies exploring genetic associations in human diseases have flourished in the past few years due to the progress of molecular biology techniques. Presently, these genomic studies focus mainly on single nucleotide polymorphisms (SNPs) as evidenced by the recent advent of genotyping chips which can analyze up to 500,000 SNPs simultaneously in a single individual. In these studies, the standard comparisons between patients and controls are performed at the level of the SNPs and at the level of their combinations which are called haplotypes. Haplotypes are of great interest for genetic association studies since they correspond to chromosomal fragments transmitted from one generation to the next. The importance of haplotypes is emphasized by the HapMap project which identifies the most prevalent and relevant haplotypes in the human population [1,2].

Normal genotyping (based on PCR/sequencing) of an autosomal SNP yields the two alleles present on the maternal and paternal chromosomes. As a consequence, SNP haplotypes cannot be determined directly because it is not known which alleles lie on the maternal chromosome and which lie on the paternal chromosome. The experimental determination of haplotypes is very expensive and time-consuming [3-5]. As an alternative, computational methods can resolve the haplotypes in a population when the genotypic information is available for enough individuals in that population (i.e the alleles present for each SNP in a genetic locus). These methods are possible because experience shows that there are a relatively small number of haplotypes present in a given population and they are maintained according to rather simple rules in the course of evolution.

In the past decade, several algorithms have been developed for inferring the haplotypes from a population of genotypes. These computational methods are either combinatorial (focus on haplotype pairs for each individual) or statistical (focus on the haplotype frequencies in the population).

An initial combinatorial method was introduced by *Clark *[6]. This algorithm first constructs a list of all haplotypes found from unambiguous individuals, i.e. individuals with at most one heterozygous site. Then, for all the ambiguous individuals (with more than one heterozygous site), it picks up a compatible haplotype from that list and adds the complementary haplotype to continue the process. This method is a variation of the parsimonious approach which asserts that the smaller the haplotypes set is to solve all the individuals, the better the solution will be. This method has two caveats: the presence of unambiguous individuals is mandatory and the final result depends on the order of treatment of the individuals. Several other authors have looked further for a parsimonious approach to extract the smallest haplotypes set explaining the genotypes in a population. For example, *Wang & al *used a "branch & bound" approach [7] and *Gusfield *a linear programming formulation [8,9] to find the most parsimonious haplotype sets count among all the possible sets of haplotypes. To take into account the haplotypes with a common evolutionary history, *Gusfield *proposed a refinement of the parsimonious principle [10] with a focus on the number of mutation events needed to generate the haplotype set rather than on the number of different haplotypes needed to resolve all genotypes in the sample. The resulting algorithm seeks a set of haplotypes that fits a perfect phylogeny. The software HAP2 has been developed based on this principle [11].

Whilst the combinatorial methods have proved valuable, generally they cannot handle a large number of SNPs (generally limited to less than 20), and many cases of missing data may prevent the resolution of haplotypes. Further, there is the theoretical problem that the true set of haplotypes carried in the population may not be the most parsimonious.

Statistical methods consider the haplotype inference problem through the distribution of the haplotype frequencies in the population rather than through the direct assignment of haplotype pairs for each individual. This statistical framework can handle a higher level of complexity in the data such as a larger number of SNPs, missing data, or multi-allelic sites. One of the best-known approaches is the EM algorithm [12] which estimates haplotype frequencies by maximizing the likelihood of the sample genotype under the assumption of Hardy-Weinberg equilibrium. The most frequent haplotypes pairs can then be assigned for each genotype in the sample. This method works well but has limitations linked to storage requirements because the number of possible haplotypes grows exponentially with the number of loci treated. A computational strategy has been proposed to alleviate this limitation by partitioning the dataset into smaller subsets for which the EM algorithm is applied and then joining the blocks of results obtained on each subset : it is called PL-EM [13,14]. As the EM algorithm often fails to capture the haplotype diversity of a sample population, alternative approaches based on Bayesian statistics have been developed [13,15]. They rely on a Gibbs sampler which computes the posterior distribution of the haplotype frequencies given the genotype of the sample and assumed prior information about the haplotype distribution [16]. These Bayesian algorithms differ in the prior they use. *Stephens & Donnelly *use an approximate coalescent prior that will give a better weight to the haplotypes that are most similar to (case of Phase1.0 [15] and HAP [17]) or that are a mosaic of (case of Phase2.1 [18]) the previously sampled haplotypes. *Niu & Al *[13] use a Dirichlet prior that chooses randomly among all possible haplotypes if the genotype cannot be made with previously sampled haplotypes. The accuracy of the statistical approaches was studied by several authors and although there is some dispute [19], it seems that the Phase algorithm provides a slightly better haplotype inference than the other methods [20-22]. However, Phase still has longer run-times. Recently, new programs such as GERBIL [23], FastPhase [24], HaploRec [25] and 2SNP [26] allow to infer haplotypes under various models of cluster of similarity in order to handle large SNP datasets. Whilst they are faster, they seem to be less accurate than Phase.

In practice, statistical methods now allow inference of haplotypes despite missing data and provide a probability for each haplotype resolution.

In this work, we present a new haplotyping algorithm which runs faster than FastPhase in common SNP datasets (less than 100 SNPs) while providing similar or better accuracy than Phase.

## Algorithm

### Rationale of the algorithm

The major hurdle for the haplotype inference problem is the very large number of haplotype pairs to be explored consistent with the genotypes in the population. Our rationale has been to try and limit the set of possible solutions to be explored, and then adapt the most efficient haplotyping procedures to this restricted set.

The algorithm that we have developed is based on 4 improvements : 1. The use of a iterative multiallelic EM (IEM) to obtain very fast EM estimations. 2. The use of a bootstrap approach to generate sufficient diversity while defining a limited set of possible haplotype pairs for each genotype. 3. The adaptation of the best haplotyping procedures to this restricted haplotype space. In particular, we have tried hereafter to estimate frequencies on this restricted haplotype space by a pseudo Gibbs sampler based on a recombination and/or a coalescent model similar to the approach proposed by *Stephens & Donnelly *[15,18]. 4. The use of a specific partition-ligation strategy to adapt our method for larger SNP datasets.

**Figure 1:**
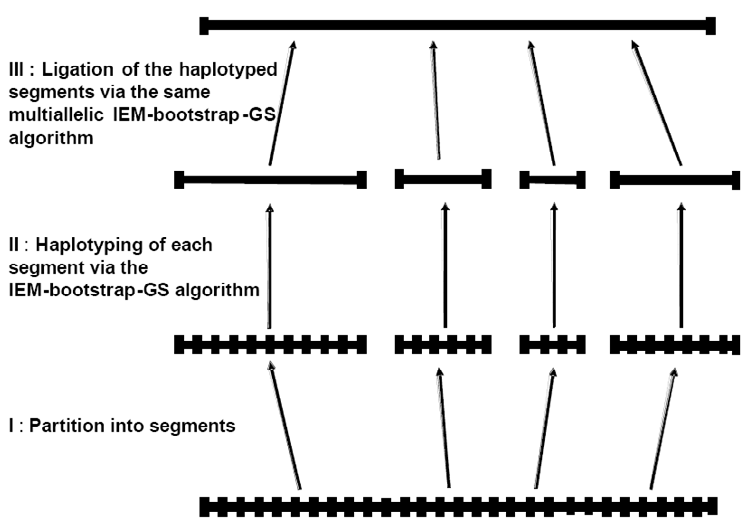
A schematic representation of the algorithm : (I) Partition strategy of the SNPs into segments thank's to the multiallelic IEM, with a new segment creation at each orphan haplotype (see text). (II) IEM-bootstrap-GS algorithm to obtain reliable haplotypes for each segment. (III) Ligation of the haplotyped segments with the same multiallelic IEM-bootstrap-GS to obtain reliable results on all the genotype dataset.

The two first improvements (IEM and bootstrap) are combined to generate a set of candidate haplotypes of reasonable size very rapidly, and then the third improvement is used to produce an optimal solution from the previously defined set of possible solutions. In case of larger datasets, we have adapted a new partition-ligation strategy in which the segments which have been haplotyped according to our algorithm (IEM bootstrap followed by a pseudo Gibbs sampler) are in turn treated as simple loci with the same multiallelic IEM, bootstrap and pseudo Gibbs sampler approach (Figure 1).

### First step: bootstrapping the iterative multiallelic EM algorithm

#### Multiallelic Iterative Expectation-Maximization algorithm (IEM)

The storage requirement and the computational effort needed by the classical EM algorithm to reconstruct the haplotype grow exponentially with the number of heterozygous and missing sites included in the genotype. In the case of multi-allelic polymorphisms this complexity easily becomes intractable for standard computers. An idea to break this growing complexity is to construct the haplotypes space gradually rather than only once. A simple iterative process constructs the haplotypes starting from 2 loci, then adding a 3^rd ^locus, then a 4^th ^locus etc... The iteration at the Lth SNP is performed by applying the EM algorithm on the haplotypes obtained at the L-1 locus combined with the alleles of the Lth locus. More precisely, the algorithm runs as follows :

Define the treatment order of the loci (random or same as the input data for example). For each genotype, take the alleles observed at the first locus as corresponding haplotype pairs. Loop until completion :

*1. Set as current locus the following one according to a defined treatment order*.

*2. Extend haplotype pairs for each genotype by combining them with the alleles observed at the current locus*.

*3. Estimate by EM the probability of each haplotype pair*.

*4. Remove all haplotype pairs whose probability is under a settable threshold (default set to 0.001)*.

Practically, we have observed that there are rarely more than 3 or 4 haplotype resolutions with a significant probability (>1%) obtained for a given subject at each iteration. This justifies keeping only a limited number of haplotypes in memory at each new locus inclusion. It is noteworthy that this approach is limited by the fact that the more SNPs there are, the more genotypes will correspond to orphan haplotypes (ie: haplotypes found in only one subject in the population). Indeed, at a given SNP inclusion, the resulting configurations for a genotype may correspond only to orphan haplotypes. In this case, the EM algorithm chooses randomly -and likely erroneously- a "most probable" configuration among them. This limitation is inherent to the EM algorithm itself. We will see that for the IEM, it needs to be addressed only for larger SNP datasets: we describe our solution in the third step below.

IEM presents a clear advantage over the classical EM approach with respect to minimizing orphan haplotypes since only the most frequent sub-haplotypes obtained with the already treated SNPs are used for defining the possible haplotypes on the next round (i.e. when adding the next SNP).

#### Bootstrap procedure

The main idea of this approach is to apply the IEM algorithm repeatedly on bootstrap samples of the original population to define the most probable corresponding haplotype pairs with more flexibility than with a single run of the IEM algorithm. This bootstrap approach introduces more possibilities in the haplotype configurations consistent with the genotypes in the population in order to increase the chance of capturing the true ones. Another advantage of the bootstrap procedure is that we use loci ordered randomly at each sampling and this allows us to escape the bias of IEM linked to the treatment order of the loci. Indeed, when running IEM in a given order one will always find the same solution, but changing the order can lead to different solutions thus to more diversity. Of course, this generation of diversity is mainly targeting rare haplotypes since frequent haplotypes are always retrieved whatever the initial order of the loci.

Here is a description of the bootstrap procedure:

Start with a single run of the IEM on the initial population in order to store the obtained haplotypes pairs for each of the genotypes. And then repeat the following step N times (N is a parameter set by default):

*1. Generate a bootstrap sample by sampling with replacement from the original sample*.

*2. Use IEM to reconstruct the haplotypes for the generated bootstrap sample with a random input order of loci*.

*3. Store the haplotype pairs obtained for each genotype included in the bootstrap sample, and their associated probability*.

*Finally, compute a posterior probability for all the haplotype pairs found. This is the sum of the probabilities stored during the bootstrap procedure divided by the number of time the genotype was sampled. At the end, for each genotype, the haplotype pairs with very low average probability (default set to less than 0.0001) are removed*.

The underlying idea of this bootstrap approach is to create enough diversity in haplotypes configurations by (1) randomizing the treatment order of loci for IEM and (2) perturbing the genotype composition of the population. On the one hand, taking multiple bootstrap samples of the population introduces more perturbations in the resulting haplotypes configurations for genotypes corresponding to rare haplotypes than for those corresponding to frequent haplotypes, and it is the rare genotypes for which the haplotyping algorithms normally diverge the most. On the other hand, applying the IEM on each bootstrap sample with random ordered loci allows building up haplotype configurations following different scenarios of dealing with frequent haplotypes.

### Second step: application of an accurate haplotype inference method on the restricted haplotype space

The bootstrapped IEM provides each genotype of the population with a limited set of candidate haplotype pairs. The initial problem thus becomes much less complex since the set of solutions to explore is smaller. On this pretreated problem, it is possible to apply sophisticated haplotype inference methods. In the present work, we have chosen the same approaches as the Phase1.0 and Phase2.1 programs [15,18] based on a pseudo-Gibbs sampler combined with various models of haplotype distribution. The two models considered rely on different models of the population evolution : (1) in the coalescence model, the future sampled haplotypes tend to be similar to the ones previously found [15,27] and (2) in the recombination model, the future sampled haplotypes tend to be a mosaic of the ones previously found [18,28]. The implementation of the pseudo Gibbs sampler is as follows :

Make a randomly ordered list of the genotypes of the population and randomly assign to each genotype one candidate haplotype pair from those selected by the bootstrapped IEM. Then, iterate a large number of times the two following steps:

*1. Update model parameters. For the coalescence model, order the list of the genotypes randomly. For the recombination model, invert the order of two randomly chosen genotypes and estimate recombination rates in view of the current haplotype assignments*.

2. For each genotype in the list :

*a. Calculate a probability according to the model for each of the haplotype pairs retained by the bootstrapped IEM under the assumption that all the others genotypes are correctly reconstructed (i.e. the assigned haplotype pairs is the true one)*.

*b. Assign to the genotype a haplotype pair from those retained by the bootstrapped IEM by a random draw according to probabilities computed in (a)*.

*The algorithm iterates steps 1. and 2. a large number of times to get sufficiently close to the final solution (burn-in iterations). For each additional iteration, current haplotype frequencies and haplotype pair probabilities are stored in order to provide reliable statistical results at the end of the iterations*.

In the following, similarly as Phase, we have compiled our program in two versions, Ishape1 for the use of the pseudo Gibbs sampler with a coalescent model (Phase1.0), Ishape2 for the use of the pseudo Gibbs sampler with a recombination model (Phase2.1). Like some other haplotyping software, ISHAPE will produce a list of haplotype pairs with a probability for each genotype and a list of the haplotypes found with their frequencies.

In practice, we find that datasets above 30–40 SNPs generate an explosion of the candidate haplotypes generated after the IEM bootstrap procedure (see the discussion on orphan haplotypes in the description, of the 1^st ^step). As a consequence, we investigated a partition-ligation (PL) approach. The strategy was as follows:

### Third step: partition-ligation strategy

Larger SNP datasets are divided into segments of limited size to avoid an explosion of candidate resolutions (Figure 1).

In each segment, candidate haplotype configurations are then generated with the Bootstrap-IEM approach and, among them, the Gibbs sampler estimates the most probable ones according to the chosen model. The iterative aspect of the Bootstrap-IEM approach adapts nicely to a progressive strategy [13] of ligating the haplotype resolutions previously found on the individual blocks. Indeed, if the resolutions on each segment are considered as a multi-allelic marker, it is possible to apply exactly the same Bootstrap-multiallelic IEM approach to delimit a set of candidate haplotypes on the whole segment dataset and so to precisely estimate haplotypes with Gibbs sampler on this limited haplotype space. To summarize, this partition-ligation strategy is done in only two steps: (1) obtain reliable solutions for each segment and (2) ligate them with the same approach applied to the pre-haplotyped segments.

We have investigated the optimal division of the SNP dataset into segments: based on a given size (10, 15, 20, 25, and 30 SNPs), or divisions into 3, 4, 5, or 6 segments. We also tested a strategy defining segments according to the proportion of orphan haplotypes generated at each iteration of the IEM approach (see the discussion on orphan haplotypes in the description of the 1^st ^step of the algorithm). When too many orphan haplotypes were generated, we backtracked and started to define a new segment starting from the SNP at stake. In other words, the segments are defined as the largest SNP subset in which the IEM generates a minimum number of orphan haplotypes. In practice, the size of the segments varies from 10 to 40 SNPs, depending on the level of linkage disequilibrium between the SNPs in the studied region. This latter PL strategy was used in the present study.

## Results

### Impact of the number of bootstrap resamplings

**Figure 2:**
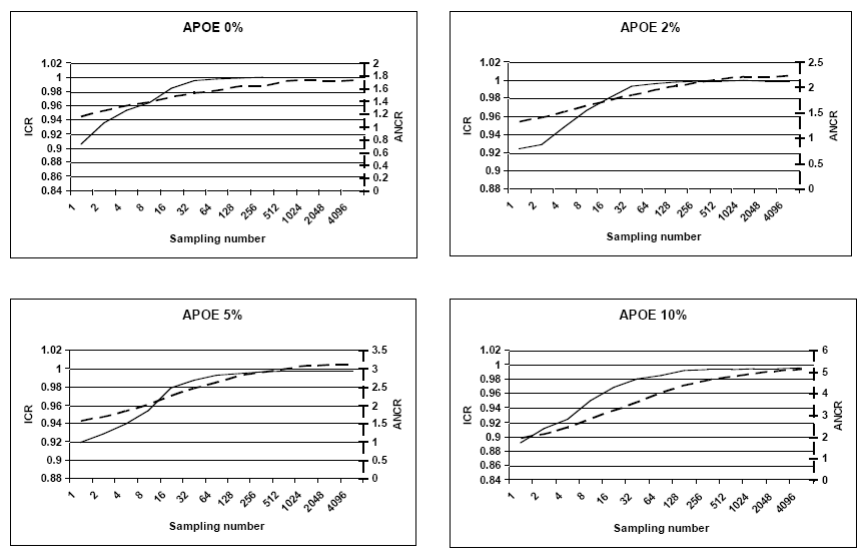
Measure of the impact of the number of bootstrap samples on the size and the relevance of the candidate haplotypes space for the APOE gene. Black line and left scale are for ICR (capture rate of true haplotypes configurations). Dashed line and right scale are for ANCR (average number of candidates per genotype).

First, we evaluated the impact of the number of bootstrap resamplings on the size and the relevance of the space of the candidate haplotypes produced. To characterize the relationships between these points, we applied our algorithm several times with a growing number of bootstrap resamplings (2, 4, 8, 16, 32, 64, 128, 256, 512, 1024, 2048 and 4096) on two datasets, GH1 and APOE (see Materials and Methods section) with different levels of missing data (0%, 2%, 5% and 10%). For each, we replicated the test 100 times and measured the ICR and the number of candidate haplotype configurations. Figure 2 summarizes the results obtained for the APOE gene. We observed a limit in the size of the haplotype space and ability of the bootstrap to capture the true haplotypes configurations was reached after 256 samplings whatever the percentage of missing data (see ICR curve in Figure 2). On our test platform (see Materials and Methods), 512 bootstrap resamplings took 0.3 and 0.7 seconds for 0% and 10% missing data (respectively).

The results were identical with the GH1 gene (data not shown). In the remainder of this work, we have thus parametered our software to perform 500 bootstrap resamplings by default.

### Relevance and size of the haplotype space generated by bootstrap resampling

To test the ability of our bootstrap approach to define a relevant haplotype space, we compared the capture rate of true haplotype configurations at different missing data levels with those obtained by the other algorithms. As shown in Table 1A, the capture rate was better for the multiallelic IEM-bootstrap than for Phase1.0, Phase2.1 on both the APOE and the GH1 datasets, and all these approaches greatly outperformed PL-EM and fastPhase. This fact suggests that applying the Phase algorithms on the restricted number of possibilities produced by the IEM-bootstrap should lead to the same accuracy, but much more quickly.

**Table 1:** capture rate and number of haplotypes detected by the various algorithms

1A :									
		0% MD		2% MD		5% MD		10% MD	

Ishape	APOE	**1.00**	32.7	**1.00**	43.6	**1.00**	53.4	**0.99**	70.4
Phase2.1		**1.00**	29.0	**1.00**	32.5	0.99	35.4	**0.99**	40.1
Phase1.0		0.99	29.0	0.99	27.8	0.99	29.6	0.98	32.5
FastPhase		0.89	29.0	0.86	27.2	0.82	27.2	0.77	27.7
PL-EM		0.89	20.0	0.90	21.1	0.89	21.3	0.88	21.1

Ishape	GH1	**0.99**	101.5	**0.99**	148.2	**0.98**	229.6	**0.97**	365
Phase2.1		0.98	71.0	0.97	70.5	0.97	74.3	0.96	83.2
Phase1.0		0.97	46.0	0.96	50.2	0.95	54.1	0.93	59.7
FastPhase		0.88	55.0	0.87	52.4	0.82	53.1	0.76	54.2
PL-EM		0.91	41.0	0.90	42.4	0.89	43.4	0.86	42.5

1B :									

	APOE (9 SNPs)				GH1 (14 SNPs)				

	0%	2%	5%	10%	0%	2%	5%	10%	

Average number of **possible **configurations per genotype	3.25	4.48	8.21	22.89	9.62	18.69	48.72	244.13	
Average number of **candidate **configurations per genotype + (ICR)	1.59 (1.0)	2.3 (1.0)	3.0 (1.0)	4.8 (0.99)	2.3 (0.99)	3.31 (0.99)	5.4 (0.98)	10.2 (0.97)	

A) Comparison of the algorithms'performance on the APOE and GH1 datasets regarding the average ICR (left column) and the average number of detected haplotypes (right column). For each level of missing data (0%, 2%, 5%, 10%), 100 experiments were performed. Best performances are highlighted in bold.

B) Estimation of the reduction of the space of possible haplotypes for the GH1 and APOE genes operated thanks to the Bootstrap-IEM. The values are the average of 100 experiments. In the lower line, the values in brackets give the correspondingaverage ICR.

Another interesting point was to compare the size of the possible haplotypes space and the candidate haplotypes space in order to have an idea of the savings of our approach in terms of computational cost. To illustrate how much the haplotype space is reduced, we compared the average number per genotype of candidate haplotypes generated by the IEM-bootstrap algorithm versus the average number per genotype of possible haplotypes, for the two real datasets GH1 and APOE with 0, 2%, 5% and 10% levels of missing data (Table 1B). In the context of complete data, we can see that with the Bootstrap-IEM, the number of resolutions for a gene of 9 SNPs (APOE) is roughly divided by 2 compared to the possible configurations, and divided by 4 for 14 SNPs (GH1), while capturing respectively 100% and 99% of the true configurations (Table 1B). The reduction is even more important when there are missing data: the haplotype space is divided by up to 5 and up to 25 for respectively APOE and GH1 with 10% missing data while keeping a capture rate above 97% (Table 1B). This suggests that our approach is more effective in the context of missing data.

Overall, in less than a second (data not shown), the IEM bootstrap approach reduces the space of haplotypes to explore by a factor between 2 and 25 compared to the regular haplotyping algorithms which must explore all possibilities, thus saving substantial computer time-costs.

### Comparison with the existing haplotyping programs

We compared Ishape to other reference software: Phase1.0 [15], Phase2.1 [18], FastPhase [24] and PL-EM [14]. All of these programs were set up with default parameters. We made the choice to use Phase both with its original coalescence model (Phase1.0) and with its recent recombination model (Phase2.1) to see how the performance of these models is influenced by the input haplotype space. To obtain reliable comparisons of the time consumption with our Gibbs sampler-based algorithms, we set the number of burn-in and main iterations to 100 to match the default for Phase. We compared our software on the experimentally determined haplotypes of GH1 and APOE datasets with various levels of missing data (see Material and Methods). For each dataset, we estimated the accuracy of the algorithms with the IER coefficient and measured the time consumption (see Materials and Methods).

We did not include HAP, HAP2, and GERBIL in the comparisons because they were shown to be less accurate than Phase [21,25]. Among the software developed to treat large numbers of SNPs, we did not test HaploRec because it currently does not handle missing data. However, we included 2SNP [26] because it was described very recently and tested only on very large datasets (generally more than 1000 SNPs).

The means obtained on 100 experiments for each level of missing data are presented in Table 2 for the real GH1 and APOE datasets. The results demonstrate that our algorithm significantly outperforms PL-EM and FastPhase for accuracy in these real datasets, and is even slightly better than Phase (Table 2A and 2B). In terms of time, Ishape2 run 6 times faster than Phase2.1 for GH1 and 4 times faster for APOE.

**Table 2:** performance of the various algorithms on the GH1 and APOE datasets

**2A. GH1 dataset**								
Soft	MD	IF	IER	Time (sec.)	MD	IF	IER	Time (sec.)

Ishape1	0%	0.927 +/- 0.001	0.119 +/- 0.001	0.9	5%	0.915 +/- 0.002	0.164 +/- 0.004	1.7
Ishape2		**0.937 +/- 0.001**	**0.103 +/- 0.001**	9.2		**0.927 +/- 0.002**	**0.147 +/- 0.004**	11.5
Phase2.1		**0.937 +/- 0.001**	**0.103 +/- 0.001**	62.9		0.924 +/- 0.002	0.148 +/- 0.004	71.4
Phase1.0		0.926 +/- 0.002	0.119 +/- 0.002	15.6		0.915 +/- 0.003	0.164 +/- 0.005	26.0
FastPhase		0.928 +/- 0.001	0.105 +/- 0.001	139.1		0.920 +/- 0.002	0.170 +/- 0.004	138.9
PL-EM		0.915 +/- 0.001	0.116 +/- 0.000	0.3		0.890 +/- 0.003	0.171 +/- 0.003	3.2
2snp		NA	0.157 +/- 0.000	**< 0.1**		NA	0.214 +/- 0.002	**< 0.1**

Ishape1	2%	0.922 +/- 0.001	0.137 +/- 0.003	1.2	10%	0.905 +/- 0.002	0.208 +/- 0.004	2.8
Ishape2		**0.933 +/- 0.001**	**0.120 +/- 0.002**	10.6		**0.916 +/- 0.002**	**0.195 +/- 0.005**	14.6
Phase2.1		0.931 +/- 0.001	0.122 +/- 0.003	64.6		0.914 +/- 0.002	0.196 +/- 0.005	82.5
Phase1.0		0.921 +/- 0.002	0.138 +/- 0.003	20.7		0.903 +/- 0.003	0.211 +/- 0.005	33.9
fastPhase		0.924 +/- 0.001	0.134 +/- 0.004	147.5		0.907 +/- 0.002	0.241 +/- 0.006	134.6
PL-EM		0.913 +/- 0.003	0.140 +/- 0.003	1.0		0.854 +/- 0.004	0.225 +/- 0.005	12.6
2snp		NA	0.176 +/- 0.002	**< 0.1**		NA	0.283 +/- 0.004	**< 0.1**

**2B. APOE dataset**								

Soft	MD	IF	IER	Time (sec.)	MD	IF	IER	Time (sec.)

Ishape1	0%	0.946 +/- 0.001	0.062 +/- 0.001	0.2	5%	**0.932 +/- 0.003**	0.109 +/- 0.005	0.4
Ishape2		0.941 +/- 0.001	0.057 +/- 0.001	3.5		0.926 +/- 0.003	**0.102 +/- 0.005**	4.1
Phase2.1		0.940 +/- 0.001	**0.055 +/- 0.001**	14.0		0.923 +/- 0.003	**0.102 +/- 0.005**	15.8
Phase1.0		**0.947 +/- 0.001**	0.062 +/- 0.000	2.7		**0.932 +/- 0.003**	0.108 +/- 0.005	3.9
fastPhase		0.876 +/- 0.001	0.118 +/- 0.002	49.1		0.870 +/- 0.003	0.181 +/- 0.005	44.2
PL-EM		0.897 +/- 0.000	0.125 +/- 0.000	0.1		0.883 +/- 0.004	0.159 +/- 0.005	0.4
2snp		NA	0.200 +/- 0.000	**< 0.1**		NA	0.227 +/- 0.004	**< 0.1**

Ishape1	2%	**0.942 +/- 0.002**	0.078 +/- 0.003	0.3	10%	**0.917 +/- 0.004**	0.149 +/- 0.007	0.6
Ishape2		0.935 +/- 0.002	**0.070 +/- 0.003**	3.9		0.910 +/- 0.004	**0.143 +/- 0.007**	4.6
Phase2.1		0.933 +/- 0.002	0.072 +/- 0.003	14.8		0.907 +/- 0.004	0.146 +/- 0.007	17.4
Phase1.0		0.941 +/- 0.002	0.078 +/- 0.003	3.2		**0.917 +/- 0.004**	0.150 +/- 0.007	5.1
fastPhase		0.875 +/- 0.002	0.140 +/- 0.003	47.0		0.864 +/- 0.004	0.225 +/- 0.007	45.4
PL-EM		0.894 +/- 0.003	0.137 +/- 0.003	0.2		0.854 +/- 0.005	0.191 +/- 0.006	1.3
2snp		NA	0.208 +/- 0.002	**< 0.1**		NA	0.259 +/- 0.004	**< 0.1**

Different missing data levels are tested, each with 100 experiments. The mean accuracy (IF and SER) and runtime of the haplotyping algorithms are compared on A. the GH1 dataset, B. the APOE dataset. The 95% confidence intervals are also given. Best performances are highlighted in bold. For 2-SNP, the software does not provide haplotype frequency estimation: thus, the IF is not available (NA).

We then tested the various programs on much larger real datasets derived from the HAPMAP project (see Material and Methods), here making a large jump from 9 SNPs (APOE) and 14 SNPs (GH1) to 80 SNPs. The results are given as an average of 100 experiments for each size of SNPs tested : 10, 20, 30, 40...80 SNPs. 2 types of SNPs subsets were analyzed: adjacent SNPs and SNPs spaced by 5 kb in average.

Table 3 summarizes the results obtained with each sofwtare on adjacent and on 5kb-spaced SNPs. Additional file 1 in supplementary material online presents the detailed results obtained for each size of SNPs : 10, 20, ...80.

**Table 3:** Accuracy and time comparison of the algorithms on the HapMap data.

	Contiguous SNPs				Spaced by 5 kb			
Software	Average SER (%)	Median SER (%)	Average ranking	Average time (sec)	Average SER (%)	Median SER (%)	Average ranking	Average time (sec)

FastPhase	1.31 +/- 0.16	0.68	2.81 +/- 0.14	100.4	3.98 +/- 0.30	2.99	2.79 +/- 0.12	88.8
Ishape1	1.40 +/- 0.16	0.63	2.87 +/- 0.15	5.0	4.88 +/- 0.36	3.51	3.99 +/- 0.15	12.3
Ishape2	**1.10 +/- 0.14**	**0.51**	**1.89 +/- 0.10**	34.9	3.60 +/- 0.29	**2.48**	**2.01 +/- 0.09**	66.1
Phase1.0	1.39 +/- 0.16	0.68	2.80 +/- 0.15	52.2	4.92 +/- 0.36	3.53	4.04 +/- 0.15	142.5
Phase2.1	1.17 +/- 0.14	0.58	2.21 +/- 0.13	215.0	**3.57 +/- 0.27**	2.53	2.11 +/- 0.10	702.0
PL-EM	1.81 +/- 0.22	0.85	3.87 +/- 0.18	6.7	5.88 +/- 0.42	4.27	5.02 +/- 0.16	5.8
2snp	1.77 +/- 0.15	1.20	4.31 +/- 0.19	**0.1**	4.71 +/- 0.28	4.01	4.24 +/- 0.17	**0.1**

Different size of SNP datasets are tested under two assumptions for the choice of the SNPs retained: adjacent SNPs and SNPs spaced by 5 kb in average. All the SNPs have a MAF above 1%. For each given size 10, 20, 30, 40, 50, 60, and 80 SNPs, one hundred different SNPs datasets were tested. The Table provides a summary showing the average performances obtained over entire HAPMAP segments tested. The 3^rd ^column presents the average rank i.e. the mean of the ranks given to each software regarding the SER they obtained for each experiment. The last column gives the average time obtained for the experiments. 95% confidence intervals are provided for the SER and for the ranking (total of 700 experiments). Best performances are highlighted in bold.

For adjacent SNPs, we saw that Ishape2 outperformed all the other software tested, even Phase2.1, at the level of the mean SER and median SER per experiment. This excellent performance was outlined by the better average ranking of the software (Table 3): overall Ishape2 had a better average rank than all the other software. For time consumption, Ishape2 runs from about 4 times faster than Phase2.1 for 10 SNPs and up to 7 times faster for 80 SNPs (see Additional file 1, supplementary material online).

For the 5kb-spaced SNPs, Phase2.1 yielded slightly better results than Ishape2 in terms of average SER but in terms of median SER and ranking, ishape2 was slightly better (Table 3). This suggests that for a few datasets, Ishape2 makes larger errors than Phase2.1. Ishape2 was faster than both Phase1.0 and Phase2.1, and could compute haplotypes up to 13 times faster than Phase2.1 on 80 SNPs with a similar accuracy (see Additional file 1, supplementary material online).

PL-EM and Ishape1 are much faster than Ishape2 but much less reliable. In this line, as for the Phase programs, the use of a recombination model appears to improve the quality of the haplotyping significantly.

Since we have tested our program on middle-size groups (60, 90 and 150 subjects), we tried to evaluate the impact of smaller and larger numbers of subjects. We used the experimental data proposed by Rieder et al. [29] regarding 11 subjects and 49 SNPs, and by Daly et al. [30] regarding 258 subjects and 103 SNPs. Table 4 summarizes all the results. For 11 subjects and 49 SNPs, 2SNP yields the best results, for 258 subjects and 103 SNPs, Ishape2 yields the best results. In this latter case, FastPhase also yields good results but runs 5 times faster than Ishape2.

**Table 4:** Accuracy and time comparison of the algorithms on four real datasets involving different numbers of genotypes

	**ACE**		**APOE**		**GH1**		**Chr 5q31**	
	**11 **genotypes		**80 **genotypes		**153 **genotypes		**258 **genotypes	

Prog	SER	Time (sec.)	SER	Time (sec.)	SER	Time (sec.)	SER	Time (sec.)

Ishape1	0.0190 +/- 0.002	0.8	0.055 +/- 0.001	0.2	0.065 +/- 0.003	0.9	0.0473 +/- 0.001	512
Ishape2	0.0184 +/- 0.0006	4.96	0.050 +/- 0.005	3.5	**0.052 +/- 0.004**	9.2	**0.0451 +/- 0.001**	5744
Phase1.0	0.0186 +/- 0.001	4.82	0.055 +/- 0.001	2.7	0.065 +/- 0.004	15.6	0.0657 +/- 0.002	21536
Phase2.1	0.0175 +/- 0.000	23.25	**0.049 +/- 0.005**	14	**0.052 +/- 0.003**	62.9	0.0501 +/- 0.001	61789
fastPhase	0.0182 +/- 0.001	37.61	0.103 +/- 0.009	49.1	0.056 +/- 0.003	139.1	0.0452 +/- 0.001	986
PL-EM	0.0573 +/- 0.005	0.51	0.165 +/- 0.000	0.11	0.060 +/- 0.004	0.31	0.0601 +/- 0.001	6507
2snp	**0.0116 +/- 0.000**	**<0.1**	0.230 +/- 0.000	**< 0.1**	0.074 +/- 0.000	**< 0.1**	0.0513 +/- 0.000	**3**

Accuracy and time comparison of the algorithms on the four real datasets ACE [29], APOE [36], GH1 [20,35] genes and data from Chr. 5q31 [30]. The 95% confidence interval corresponds to 100 runs of each program for ACE, APOE and ACE, and to 10 runs for Chr.5q31 data. Best performances are highlighted in bold.

## Discussion and conclusion

In this work, we have presented new software (Ishape) for the computation of haplotypes. This software relies on the combination of the following improvements: 1. Use of a iterative multiallelic EM algorithm; 2. Use of a bootstrap procedure; 3. Adaptation of the pseudo Gibbs sampler to a limited set of candidate haplotypes; and 4. Use of a specific partition-ligation strategy. When reviewing the literature, we found that an iterative haplotyping approach has been previously described for biallelic polymorphisms [31], otherwise all these improvements are totally new.

We performed comparison tests of Ishape with other reference programs such as Phase, Fastphase, and PL-EM. We first performed a test on 2 haplotype real datasets with or without missing data (GH1, 14SNP, 150 subjects ; APOE, 9 SNPs, 90 subjects) and found that Ishape2 and Phase2.1 yielded similar results on these "small" datasets (Table 2).

We then performed the comparisons on SNP datasets of various size (10, 20, 30, 40, 50, 60, and 80 SNPs) derived from the HapMap project. The SNPs were either adjacent or spaced by 5 Kb and all SNPs had a minor allele frequency (MAF) > 1%. To ensure a fair comparison, the parameters were set identical to the Phase2.1 default parameters (100 burn-in iterations, 100 main iterations, thin-in interval of 1). We have limited our tests on the HapMap data with up to 80 SNPs for runtime reasons and also because biologists usually work at the gene level, and few genes will contain more than 80 SNPs.

Ishape2 produced results with a similar accuracy as Phase2.1 but much more rapidly. It was also more reliable than FastPhase and just as rapid. PL-EM was not competitive with any of the other three programs in terms of accuracy.

It is interesting to note that when working on adjacent SNPs, Ishape2 outperformed Phase and FastPhase in speed and accuracy. When working on SNPs spaced by 5 kB, our data (see Additional file 1, supplementary material online) suggest that if one considered more than 100–120 SNPs, Fastphase would run faster than Ishape2.

Ishape2 behaves better in genomic regions exhibiting a certain level of linkage disequilibrium probably because the reduction of the haplotype space is more important and relevant in that case, and high LD helps the convergence of the posterior Gibbs sampler. In the case of a low level of LD, the diversity generated may become too important to ensure a proper convergence of the Gibbs sampler, and the risk of missing a true configuration increases : Ishape2 performed better in terms of median SER and average ranking, but worse in terms of mean SER for the 5kb-spaced SNPs (Table 3).

Finally, we have tested two additional real datasets with small and large numbers of subjects (respectively 11 and 258). Table 4 suggests that Ishape2 is more robust when the number of subjects is larger. This is easily understandable since Ishape2 relies on a bootstrap approach with multiple samplings in the population. If the population is too small, the samplings will not bring enough diversity. Table 4 shows again that with over 100 SNPs fastPhase becomes very competitive in terms of speed and accuracy: this warrants further studies.

The results provided by 2SNP show that it is not suited for the common use of haplotyping software by biologists (i.e. less than 100 SNPs). However one can remark that when dealing with independent SNPs, its reliability increases (Table 3) and it will be of interest to compare it with Fastphase on very large numbers of SNPs.

The model of Ishape which combines a bootstrap approach and a Gibbs sampler approach opens new possibilities for fast and accurate haplotyping. Future improvements of the software will target a better treatment of SNPs with low LD (5 Kb-spaced SNPs) and the rapid treatment of even larger numbers of SNPs. For that, we plan to refine the threshold values used, test other partition-ligation strategies, and investigate alternatives to the Gibbs sampler since this is the rate limiting step.

In terms of applications, Ishape2 appears to be a robust haplotyping program suitable for disease association studies which typically address less than 100 SNPs at a time. It may prove advantageous to use Ishape2 to compute LD in genetic regions, since it is faster and more reliable on neighboring SNPs. Ishape2 may also be useful for computing haplotypes serially on genes spanning whole genomes since this application will expand with the advent of large scale genotyping chips [32-34].

In conclusion, the results presented here show that the Ishape heuristic approach is very competitive in terms of accuracy and speed and deserves to be evaluated extensively for its future wide use.

## Methods

### Datasets

We used real datasets for which the correct haplotypes were completely or partially determined.

For three real datasets, the haplotypes were determined experimentally by molecular haplotyping techniques. Thus it was possible to compare the estimated haplotypes with the true ones:

-The GH1 dataset provided by Horan et al [20,35]. The promoter of the growth hormone (GH1) gene spans 535 bps, and is highly polymorphic with 14 loci whose minor allele frequency (MAF) is greater than 1%. It contains genotypes from 154 unrelated individuals and 38 different haplotypes based on 13 biallelic loci and 1 triallelic locus.

-The APOE dataset provided by Orzack et al [36]. It contains genotypes from 80 unrelated individuals from 3 ethnic groups: 18 Asian, 19 African and 43 Caucasians. The APOE locus is composed of 9 SNPs with MAF>1%. 17 haplotypes were identified experimentally.

-The ACE dataset provided by Rieder et al [29]. It contains genotypes from 11 unrelated individuals and 13 haplotypes were identified experimentally. The Angiotensin Converting Enzyme gene contains 49 exploitable SNPs for this study (singletons with only one variant allele were removed).

In order to test our algorithm more precisely in case of missing data, we have generated 3 sets of 100 replicates representing respectively of 2%, 5% and 10% of missing data for each of these GH1 and APOE datasets.

We also used the HapMap trios (parents and a child) [1,2] which allowed us to derive very large sets of reliable haplotypes for the parents population. We took randomly, in the HapMap CEU autosomal data, 100 replicates of 10, 20, 30, 40, 50, 60 and 80 SNPs of 60 individuals (30 trios in which we kept only the parents, not the child). We tested 2 kinds of SNPs subsets : on the one hand, consecutive SNPs in order to maximize the impact of LD in the computation of the haplotypes, and on the other hand, SNPs spaced by 5 kB in average, in order to minimize the impact of LD in the computation of the haplotypes. The SNPs chosen had a minor allele frequency (MAF) above 1%. They spanned less than 1 Mb for each replicate, and the real haplotypes could be determined simply under the assumption that no recombination event occurred during the last meiosis. This approach allowed us to resolve an average of 85% of the ambiguous sites in all the data reliably. The resolved sites were flagged in order to compare the haplotype inference software only on these sites.

Finally, we also tested the data generated by Daly et al. [30] corresponding to the genotypes of 129 trios. We worked on the parental genotypes (258 subjects) made of 103 SNPs with a MAF above 5% and spanning over 500 kb on chromosome 5q31.

### Measures of performance

We have measured the accuracy of our software on real datasets. First, we have investigated the capacity of our IEM bootstrap approach to generate enough diversity in the space of possible haplotypes to comprehend the whole variety of existing haplotypes. Second, we have compared it with the most used haplotypes inference software.

The aim of the IEM bootstrap approach is to delimit a small haplotype space which captures at best the true haplotype configurations. We thus used the three following measures: the average number of candidate resolutions per genotype provided by the bootstrap-IEM (called hereafter ANCR), the total number of haplotypes, and the proportion of individuals for whom the real haplotype pair is included in the set of candidates (called hereafter ICR for individual capture rate).

In order to compare the accuracy of the different haplotype inference software, we worked on real datasets (see below) and computed two measures, used in most of the other studies [20,21], focusing on the correct haplotype assignments for each individual of the sample. These measures are the individual error rate (IER) and the switch error rate (SER). The IER is the proportion of individuals in the sample for whom the most probable inferred haplotype pair is not correct. The SER is the proportion of ambiguous loci in which the phase is incorrectly inferred compared to the precedent ambiguous locus. This latter measure not only assesses whether the haplotype assignments are correctly made, but also determines how close are the inferred and true haplotypes configurations, in terms of switch events. In case of large data sets such as the HapMap data set we used, the SER is more relevant than the IER to estimate which haplotyping method is the best because the more loci there are at stake, the more the phased genotypes will tend to be incorrect in at least one site, and as a consequence, it becomes more interesting to look at the number of switch events needed to recover the true phase.

For the comparison of haplotype frequencies, we first calculated the true haplotype frequencies from the real data by using the gene-counting technique. Then, we computed the I_F _score previously used by Excoffier & Slatkin [12] to evaluate how close the true frequencies and the estimated frequencies are from each other.

We also ranked from 1 to 7 the seven programs tested (FastPhase, Ishape1, Ishape2, Phase1.0, Phase2.1, PL-EM, 2SNP) according to the SER derived from each experiment. Table 3 presents the average rank obtained for each program.

We have presented the 95% confidence intervals where applicable : in Table 2 for tests done on the same SNP dataset (APOE and GH1), in Table 3 for the summary of the results obtained in the HapMap data on 10, 20, ... and 80 SNPs, and in Table 4 for the mean of several experiments on the same dataset.

All the programs were run on an AMD athlon 3200 with 1 Go. of RAM to measure time consumption under the same conditions.

## Authors' contributions

C. Coulonges, O. Delaneau, and J-L Spadoni worked on developing the methods and programs used in this study under the direct supervision of J-F. Zagury who conceived the study. P-Y. Boelle and G. Nelson have had an active intellectual input in the study by providing their extensive knowledge regarding statistics and haplotyping. All the authors have read and approved the final manuscript.

## Availability and requirements

The Ishape software is available at: 

To download the software, use the login: ishape, and the password: ishape.

It is platform independent, written in c++, versions for UNIX and Windows are proposed: do not forget to read the small file, readme.txt, to get the detailed information.

The software will be freely available to academics, and a license will be needed for non-academics.

## Supplementary Material

Additional file 1 Accuracy and time comparison of the algorithms on the HapMap data according to the number of haplotyped SNPs. Different size of SNP datasets are tested under two assumptions for the choice of the SNPs retained: adjacent SNPs and SNPs spaced by 5 kb in average. All the SNPs have a MAF above 1%. For each given size 10, 20, 30, 40, 50, 60, and 80 SNPs, one hundred different SNPs datasets were tested. The 3^rd ^column presents the average rank i.e. the mean of the ranks given to each software regarding the SER they obtained for each experiment. The last column gives the average time obtained for the 100 experiments. Best performances are highlighted in bold. Click here for file
